# Mr. Chalmers, on Pneumonia, &c.

**Published:** 1807-02

**Authors:** W. Chalmers

**Affiliations:** Hythe


### *To the Editors of the Medical and Phyfical Journal.*

GENTLEMEN,

I Have not the honour of knowing your correspondent, Mr. Alexander Denmark, whose publication of a case of gastritis appeared in your Journal for this month; I should, however, from the strangeness of his language, be led to conclude he is a *naval practitioner,* even if he had not informed me.

My remarks on a case of diseased bladder, which had lately a place in one of your numbers, may probably induce some of your readers to believe me possessed of an illiberal aversion to that class of men. While I allow that they labour under many disadvantages, inseparable from their floating situation, I am sorry to advance a truth too well known, I mean their not interesting themselves in their duty, and their want of ability to perform it, which likewise seem inseparable from that part of the profession. I am far from saying those defects are universal, but I am as far from saying that they are not general in the medical department of the navy.

As

---

As to Mr. Denmark's observations on the case of Wm. Usher, who complained on the morning of the 4th of Aug. last, I think they want amendment; and as to the treatment of the patient, I conceive it to have been improper.

The symptoms related by the gentleman in question, are certainly those which indicate pneumonia. In delivering them I believe him to have been inaccurate, at least in one particular. I allude to the great velocity of pulse on the first day, which he tells us was 140, while the patient's skin continued natural.

The operation of the antimonial emetic appears to me, I must confess, in no favorable light; and the great delay in taking blood from a young man of William Usher's description, labouring under difficulty of breathing to so great a degree as to become anhelous, and attended with every febrile symptom, excites my astonishment. The small quantity taken when the lancet was at length recurred to; the culpable moderation of the operator; together with a want of direction in his attention to the other appearances of disease, sufficiently evince the necessity of being more circumspect in the diagnosis, and more rigorous in the treatment of pneumonia, than Mr. Denmark seems to have been.

I do not deny the possibility of the immense frequency of pulse on the first day, nor its amounting to 160 (as related) on the second day, "*while the surface*" might well be said to be "*warmer than natural.*" But to me the statement seems incorrect; nor can I see any one symptom by which the practitioner could have the most distant "*preconception*" (as he calls it) of gastritis, before August 6.

The exhibition of aromatics in gastritis is new; and the indulging a patient, whose dissolution was but too evidently at hand, in his solicitations of rising out of bed, cannot be viewed by the eye of humanity without horror. We are informed that the poor sufferer tottered, when he attempted to walk, on the day he first complained; was that to be wondered at? But there is a subject of wonder presents itself, when we enquire for what purpose he was suffered to try to walk; and this subject becomes more extensive on reading the following words, remarked on the 8th day of the month, "*when he returned at five o'clock he was greatly exhausted; pulse hardly perceptible, and about 160; great stupor and inclination to coma,*" &c.

Upon the whole, I am of belief that Mr. D. has mistaken the disease; that he has omitted several characteristic symptoms in his case; and, that, although some of hi his steps were judicious, and perhaps all well intended, yet, there was a necessary degree of discrimination wanting, to ensure his success on board, and the approbation of his readers. The patient might have died under the best treatment, and ablest professional talents, and possibly Mr. Denmark paid him every attention. If my sentiments do not correspond with his, in regard to the present case, I hope he will not conceive that I have taken any unfair advantage of him. I forbear commenting on his corrolaries; and in answer to the questions he proposed, beg leave to refer him to my paper on pulmonic inflammation.

Before I conclude, I embrace this opportunity of mentioning the great desire which pneumonic patients have of laying on their backs; and the frequent occurrences of slight phrenitis attending inflammation of the thoracic and abdominal viscera, even in the first stages of the disease. When pyrexia has existed for some time, and heated the mass of blood to a degree much above health, this phrenitic affection would seem concomitant, and to be expected, for then every part of the system assumes inflammatory appearances, and the disease sometimes removing in a great measure from its original seat, takes possession of some other part, in general, contiguous, or nearly related to the first, by its vessels and nerves. Accidental circumstances will likewise occur in the progress of disease, often producing the most unexpected changes.

*Hythe,* I am, &c.

*December* 4, 1806. W. CHALMERS, A. M.

